# A novel nutritional index “simplified CONUT” and the disease risk index independently stratify prognosis of elderly patients with acute myeloid leukemia

**DOI:** 10.1038/s41598-020-76250-8

**Published:** 2020-11-10

**Authors:** Hajime Senjo, Masahiro Onozawa, Daisuke Hidaka, Shota Yokoyama, Satoshi Yamamoto, Yutaka Tsutsumi, Yoshihito Haseyama, Takahiro Nagashima, Akio Mori, Shuichi Ota, Hajime Sakai, Toshimichi Ishihara, Takuto Miyagishima, Yasutaka Kakinoki, Mitsutoshi Kurosawa, Hajime Kobayashi, Hiroshi Iwasaki, Daigo Hashimoto, Takeshi Kondo, Takanori Teshima

**Affiliations:** 1 Department of Hematology, Faculty of Medicine, Hokkaido University, Sapporo, Japan; 2 Department of Hematology, Sapporo City General Hospital, Sapporo, Japan; 3 Department of Hematology, Hakodate Municipal Hospital, Hakodate, Japan; 4 Department of Hematology, Tonan Hospital, Sapporo, Japan; 5 Department of Hematology, Japanese Red Cross Kitami Hospital, Kitami, Japan; 6 Blood Disorders Center, Aiiku Hospital, Sapporo, Japan; 7 Department of Hematology, Sapporo Hokuyu Hospital, Sapporo, Japan; 8 Department of Hematology, Teine Keijinkai Hospital, Sapporo, Japan; 9 Department of Hematology, Kin-Ikyo Chuo Hospital, Sapporo, Japan; 10 Department of Hematology, Kushiro Rosai Hospital, Kushiro, Japan; 11 Department of Hematology, Asahikawa City Hospital, Asahikawa, Japan; 12 Department of Hematology, Hokkaido Cancer Center, Sapporo, Japan; 13 Department of Hematology, Obihiro Kosei General Hospital, Obihiro, Japan; 14 Department of Hematology, Sapporo Kosei General Hospital, Sapporo, Japan

**Keywords:** Acute myeloid leukaemia, Prognostic markers

## Abstract

Elderly patients aged 65 or older with acute myeloid leukemia (AML) have poor prognosis. The risk stratification based on genetic alteration has been proposed in national comprehensive cancer network (NCCN) guideline but its efficacy was not well verified especially in real world elderly patients. The nutritional status assessment using controlling nutritional status (CONUT) score is a prognostic biomarker in elderly patients with solid tumors but was not examined in elderly AML patients. We performed prospective analysis of genetic alterations of 174 patients aged 65 or older with newly diagnosed AML treated without hematopoietic stem cell transplantation (HSCT) and developed simplified CONUT (sCONUT) score by eliminating total lymphocyte count from the items to adapt AML patients. In this cohort, both the NCCN 2017 risk group and sCONUT score successfully stratified the overall survival (OS) of the elderly patients. A multivariable analysis demonstrated that adverse group in NCCN 2017 and high sCONUT score were independently associated with poor 2-year OS. Both risk stratification based on NCCN 2017 and sCONUT score predict prognosis in the elderly patients with newly diagnosed AML.

## Introduction

Elderly patients aged 65 or older with acute myeloid leukemia (AML) are often ineligible for hematopoietic stem cell transplantation (HSCT) and generally have a poor prognosis. Although this patient population represents majority of AML patients, currently used prognostic indices are mostly derived from younger patient data. Analysis of large-scale real world data of the elderly patients is warranted to confirm the efficacy of these prognostic indices. The prognostic risk classification based on national comprehensive cancer network (NCCN) Guidelines Version3. 2017; NCCN 2017^1^ is widely used; however, the impact of this classification on the prognosis of such elderly AML patients is unclear. While nutritional status assessment using controlling nutritional status (CONUT) score^2^ based on serum level of albumin (Alb), total-cholesterol (T-chol), and total lymphocyte count (TLC) has been shown to predict prognosis of elderly patients with solid tumors and hematological malignancies such as multiple myeloma and malignant lymphoma^3–8^, its prognostic significance in elderly patients with AML remains to be clarified. In the current study, we aimed to determine the prognostic value of NCCN 2017 and nutritional status in elderly patients with newly diagnosed AML.

## Patients and methods

### Patients

Hokkaido Leukemia Net (HLN) prospectively collects AML samples from hospitals of North Japan Hematology Study Group (NJHSG). In this study, we focused on newly diagnosed AML patients aged 65 or older treated without HSCT and investigated cytogenetic and molecular abnormality of leukemic cells including *FLT3-ITD*, *NPM1*, *CEBPA*, and *KIT*, as previously described^9^. The presence of *TP53* mutation was not determined in the present study. We stratified the patients into favorable, intermediate, and adverse risk groups based on NCCN 2017. A total of 174 patients aged 65 or older with AML treated without HSCT from 2010 to 2018 were enrolled in this study (Fig. S1). The study was conducted in compliance with the ethical principles based on the Declaration of Helsinki and was approved by the institutional review board of Hokkaido University Hospital (No. 015-0344). Written consent was obtained from each patient for the study participation.

### Risk indices

The CONUT score was calculated from Alb (g/dL), T-chol (mg/dL) and TLC (/μL), as previously reported^2^ (Table 1A). Since AML patients often present low lymphocyte counts^10^, we developed simplified CONUT (sCONUT) score by eliminating TLC from evaluation criteria (Table 1B). The patients with score 3 or more at diagnosis were defined as high group. We also evaluated the following nutritional index for patients with evaluable data based on previous reports. The geriatric nutritional risk index (GNRI) score^11^ was calculated as 1.489 × Alb (g/L) + 41.7 × weight (kilograms)/ideal body weight, with ideal weight was calculated according to the Lorentz equations. The prognostic nutritional index (PNI) score^12^ was calculated as 10 × Alb (g/dL) + 0.005 × TLC (/μL). For GNRI and PNI, we defined the patients with higher score than median score as high group and patients with lower score as low group. The information about the weight and height (meters) was taken on the day of admission for all patients.

**Table 1:** The calculating tables of CONUT score (A) and simplified CONUT score (B).

**(A) CONUT score**				
Alb (g/dL)	3.5–	3.0–3.4	2.5–2.9	– 2.4
	0	2	4	6
T-chol (mg/dL)	180–	140–179	100–139	– 99
	0	1	2	3
TLC (/μL)	1600–	1200–1599	800–1199	– 799
	0	1	2	3
Total score	0–1	2–4	5–8	9–12
Group	Normal	Mild	Moderate	Severe
**(B) simplified CONUT score**				
Alb (g/dL)	3.5–	3.0–3.4	2.5–2.9	– 2.4
	0	2	4	6
T-chol (mg/dL)	180–	140–179	100–139	– 99
	0	1	2	3
Total score	0–2	3–		
Group	Low	High		

*Alb* albumin, *T-chol* total cholesterol, *TLC* total lymphocyte count.

### Statistical analysis

Overall survival (OS) was calculated from the day of diagnosis until death or last follow-up. The probability of OS was estimated using a Kaplan–Meier method, and differences between patient groups were analyzed using the log-rank test. The baseline patient characteristics were tabulated to check imbalance in the demographic information. The risk factor at diagnosis for OS was evaluated by multivariable Cox regression using stepwise variable selection. Death within 2-years from the day of diagnosis was defined as censoring for the Cox regression model. The differences of the treatment regimens were evaluated by X^2^-test. All *P values* were 2-sided and a *P-value* of 0.05 was used as the cutoff for statistical significance. All statistical analyses were performed with IBM SPSS Statistics 26 software.

## Results

### Patient characteristics

**Figure 1:**
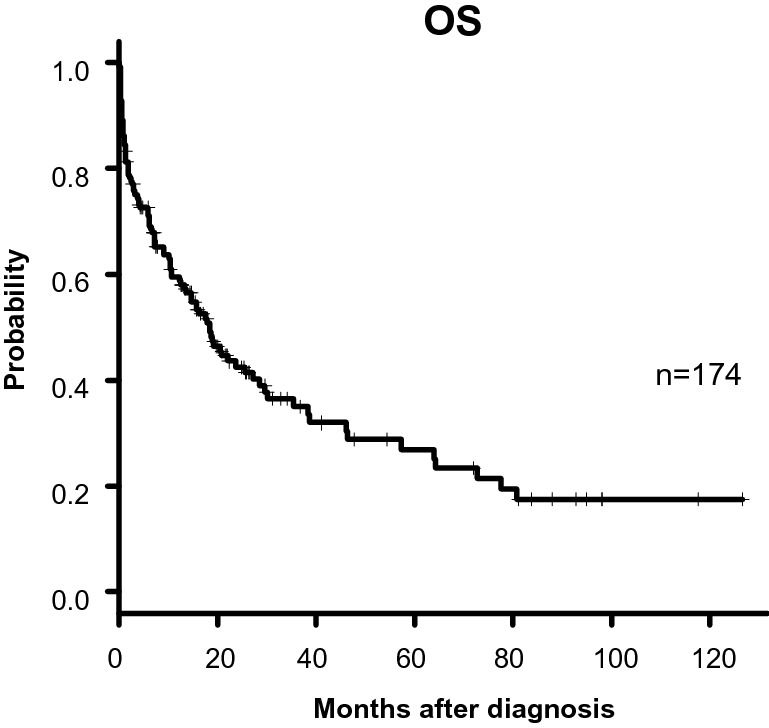
Kaplan–Meier plots of overall survival (OS) of patients for the entire cohort.

Baseline patient characteristics were listed in Table 2. A median patient age at diagnosis was 72 years, ranging from 65 to 93 years. All patients were investigated their cytogenetic and molecular abnormalities of leukemic cells, including *FLT3-ITD*, *NPM1*, *CEBPA*, and *KIT* at diagnosis. According to the NCCN 2017 stratification^1^, 22%, 58%, and 20% of the patients were classified their risk status as favorable, intermediate, and adverse, respectively. Both the body height and weight at diagnosis were available in 137 patients, and the median body mass index (BMI) was 24 ranging from 14 to 34. For blood sample test, complete blood count and differential white blood count were available in all 174 patients. The median TLC was 1.6 × 10^9^/L, which was consistent with that in a previous study of AML patients^10^. Serum albumin levels at diagnosis were available in 152 patients with 4.0 g/dL as median level, ranging from 1.8 to 5.9 g/dL. Serum total cholesterol levels at diagnosis were available in 112 patients with 140 mg/dL as median level, ranging from 81 to 248 mg/dL. According to the CONUT scores^2^, 21%, 52%, 25%, and 2% of the patients were classified as normal, mild, moderate and severe group, respectively. For sCONUT scores, 68% of the patients were classified as low group and 32% as high group. Induction therapy based on decision of each physician varied according to risk factors of the disease and general status of patients. As shown in Table 2, 86.3% of the patients were initially treated with chemotherapy. The remaining 13.2% of the patients were treated with best supportive care. Overall, 57 patients achieved CR (33%; Table 2). The median OS was 15.2 months and the 5-year OS was 24.8% (Fig. 1).

**Table 2:** Patient characteristics and univariate analysis of the risk factors associated with 2-year OS.

Characteristics	No. (%)	OS (%)	*P* value
**Sex (n = 174)**			0.155
Male	106 (60.9)	30.2	
Female	68 (39.1)	45.7	
**Age (n = 174)**			0.039
65–70	74 (42.5)	42.2	
71–80	73 (42.0)	32.3	
81–	27 (15.5)	26.6	
Median (range)	72 (65–93)		
**NCCN2017 (n = 174)**			< 0.01
Favorable	39 (22.4)	59.9	
Intermediate	101 (58.1)	41.8	
Adverse	34 (15.5)	4.4	
**BMI (n = 137)**			0.553
≥ 26	37 (28.2)	40.9	
< 26	100 (71.8)	38.6	
Median (range)	24 (14–34)		
**WBC count (n = 174)**			0.626
≥ 6.5 × 10^9^/L	86 (49.4)	37.2	
< 6.5 × 10^9^/L	88 (50.6)	34.1	
Median (range), × 10^9^/L	6.6 (0.46–126.1)		
**TLC (n = 174)**			0.146
≥ 1.6 × 10^9^/L	87 (50.0)	37.3	
< 1.6 × 10^9^/L	87 (50.0)	26.9	
Median (range), × 10^9^/L	1.6 (0.04–34.8)		
**Alb level (n = 152)**			0.067
≥ 3.0 g/dL	90 (62.0)	35.5	
< 3.0 g/dL	62 (38.0)	21.2	
Median (range), g/dL	4.0 (1.8–5.9)		
**T-Chol level (n = 112)**			0.072
≥ 140 mg/dL	56 (50.0)	35.4	
< 140 mg/dL	56 (50.0)	24.0	
Median (range), mg/dL	140 (81–248)		
**GNRI score (n = 132)**			0.822
≥ 50	66 (50.0)	26.9	
< 50	66 (50.0)	29.7	
Median (range)	50 (11–68)		
**PNI score (n = 152)**			0.866
≥ 45	76 (50.0)	32.1	
< 45	76 (50.0)	29.5	
Median (range)	45 (21–220)		
**CONUT score (n = 112)**			0.102
Normal	24 (21.4)	45.2	
Mild	58 (51.8)	41.6	
Moderate	28 (25.0)	11.1	
Severe	2 (1.8)	0.9	
**Simplified CONUT score (n = 112)**			0.018
Low	76 (67.9)	50.1	
High	36 (32.1)	9.1	
**Treatment**			0.675
IDR + AraC	52 (29.9)	42.3	
DNR + AraC	19 (10.9)	10.5	
DNR + BHAC	21 (12.1)	4.8	
CAG	25 (14.4)	16.0	
AZA	12 (6.9)	8.3	
Low-dose AraC	7 (4.0)	0.0	
Other chemotherapy	15 (8.6)	10.0	
Best supportive care	23 (13.2)	0.0	
**Response**			< 0.01
CR	57 (32.8)	36.8	
Non-CR	98 (56.3)	12.2	
ND	19 (10.9)		

*BMI* body mass index, *WBC* white blood cell, *TLC* total lymphocyte count, *Alb* albumin, *T-Chol* total cholesterol, *IDR* idarubicin, *AraC* cytarabine, *DNR* daunorubicin, *BHAC* enocitabine, *CAG* low-dose cytarabine, aclarubicin hydrochloride, and granulocyte colony stimulating factor, *AZA* azacitidine, *CR* complete remission, *ND* no data.

### The prognostic risk classification based on NCCN 2017

**Figure 2:**
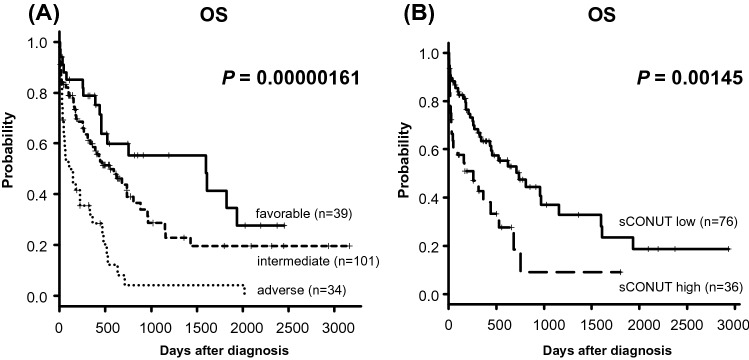
Kaplan–Meier plots of OS according to NCCN 2017 risk classification (**A**) and sCONUT score (**B**).

The risk classification based on NCCN 2017 successfully stratified the overall survival of the patients (5-year OS; favorable group, 41.5% vs. intermediate group, 22.5% vs. adverse group, 4.38%, *P* = 0.00000161, Fig. 2A).

### CONUT score and sCONUT score as a prognostic biomarker

**Figure 3:**
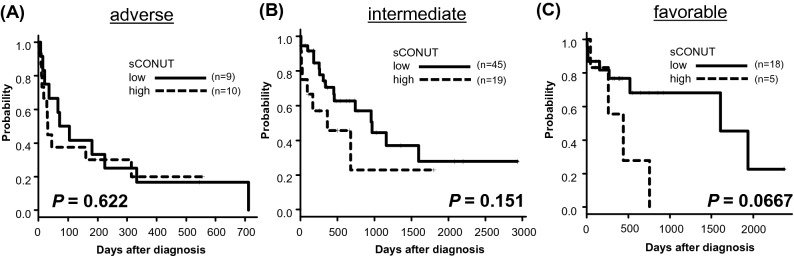
Kaplan–Meier plots of OS according to sCONUT score in patients with adverse risk (**A**), intermediate risk (**B**), and favorable risk (**C**) stratified by NCCN 2017 risk classification.

Based on the classic CONUT score classification, we found that OS was lower in moderate and severe groups, which was not significant (5-year OS; normal group, 18.6%; mild group, 22.3%; moderate group, 11.7%; severe group, 0.%, respectively, *P* = 0.0881, Fig. S2). This score did not work as a prognostic indicator with statistical significance. We hypothesized that inclusion of lymphocyte count, which was usually low in AML patients^10^, hampered to stratify prognosis in AML patients. We therefore developed the sCONUT score by simply omitting TLC from the 3 factors of the CONUT score. For sCONUT score, which stratify patients into 2 groups depend on scoring of 2 parameters, OS was significantly lower in patients with high sCONUT score than in those with low sCONUT score (5-year OS; 26.5% vs. 9.97%, *P* = 0.00145, Fig. 2B). Then, we investigated if the sCONUT score could further stratify risks of the patients in each NCCN 2017 risk group. While there were no significant prognostic impacts of the sCONUT score in patients with adverse and intermediate risk groups (Fig. 3A,B), there was a tendency in favorable risk group that patients with high sCONUT score had poorer prognosis than patients with low score (5-year OS; 42.3% vs. 0%, *P* = 0.0667, Fig. 3C). Because the poor nutritional status might be related to best supportive care option without chemotherapy, we further investigated subgroup analysis depends on treatment option. The significantly larger proportion of patients with high sCONUT score were treated without chemotherapy than the patients with low sCONUT score (33.3% vs. 8.2%, *P* = 0.0029, Fig. S3(A), S3(C)). On the other hand, we found no significant difference of the percentage of the patients treated without chemotherapy between NCCN-2017 risk groups (favorable; 18.2%, intermediate; 13.8%, adverse; 12.5%, P = 0.816, Fig. S2(D)). We additionally analyzed the outcome in patients treated with or without chemotherapy. In patients treated with chemotherapy, sCONUT score could stratify 89 patients into 2 groups with significantly different outcomes; sCONUT low (n = 67), sCONUT high (n = 22) (2-year OS, 42.1% vs. 21.6%, *P* = 0.0315, Fig. S3(E)). We found that all patients treated without chemotherapy had dismal prognosis, died within 300 days after diagnosis, and sCONUT score did not divide the prognosis (*P* = 0.152, Fig. S1(F)).

### Univariate and multivariable analyses of clinical prognostic factors

We analyzed various prognostic factors for OS (Table 2). In a univariate analysis, GNRI score, PNI score and CONUT score were not associated with poor 2-year OS. On the other hand, higher age, adverse risk group based on NCCN 2017 classification, high sCONUT score, and not reached complete remission (CR) after first induction chemotherapy were associated with poor 2-year OS. We therefore performed multivariable analysis that included higher age, adverse risk group based on NCCN 2017 classification, high sCONUT score, and non-CR. In this multivariable analysis, adverse group in NCCN 2017 risk classification, and high sCONUT score and non-CR were independently associated with poor 2-year OS (NCCN 2017; HR 3.16; 95% CI 1.87–5.36, *P* = 0.0000189, simplified CONUT score; HR 1.76; 95% CI 1.11–2.78, *P* = 0.0163; non-CR; 2.82; 95% CI 1.70–5.71, *P* = 0.0000669; log-rank, Table 3).

**Table 3:** Multivariable analysis of the risk factors associated with 2-year OS.

Characteristics	Hazard ratio	95% CI	*P* value
Age more than 80	1.65	0.94–2.90	0.08
Adverse group in NCCN 2017	3.16	1.87–5.36	< 0.01
High simplified CONUT score	1.76	1.11–2.78	0.02
Non-CR	2.82	1.70–4.71	< 0.01

## Discussion

AML occurs in all age groups and is most common in patients older than 65 years^13^. Despite the development of lower-intensity treatment due to the discovery of novel agents, there is still no standard treatment of choice for the elderly patients with high risk AML^14^. However, some elderly patients are successfully treated with intensive chemotherapy. Hence, it is crucial to find prognostic biomarkers for elderly patients with newly diagnosed AML. Most commonly used prognostic risk classification, NCCN 2017 was developed based on data of younger patients^1^. In the current study, we find that the NCCN 2017 is also feasible for elderly patients with 65 years old or older.

The nutritional status assessments using GNRI; BMI and serum level of Alb, PNI; serum level of Alb and TLC, and CONUT score; serum level of Alb, TLC and T-chol, are developed to predict tolerability to cancer treatment in the elderly patients with solid tumors^3,4,15–17^. Recently, the CONUT score has been shown to predict prognosis in patients with some hematological malignancies^5–8^. However, the prognostic value in AML remained to be determined. In the current study, we clarified that these previously developed nutritional scores did not clearly stratify survival for elderly patients with newly diagnosed AML. We therefore developed the sCONUT score by simply omitting TLC from the 3 factors of the CONUT score as mentioned above. The sCONUT scoring can predict the prognosis of the elderly patients with AML. The sCONUT score is a useful and simple risk classification that can be easily calculated based on the result of blood sample test at diagnosis independent from NCCN disease risk score based on genetic alteration. It is important to know both disease factor and host nutritional status independently affect outcome in elderly AML patients. Especially for the elderly patients, assessment of nutritional status could be prioritized than disease risk assessment, as we showed that even patients with favorable risk group by NCCN 2017 classification could be further divided by nutritional status (Fig. 3C). Additionally, we demonstrated that the larger number of patients in sCONUT high groups were treated without chemotherapy compared with patients in sCONUT low groups and in patients treated with chemotherapy, sCONUT score could stratify 89 patients into 2 groups with significantly different outcomes. These results show that sCONUT score would be useful as a prognostic biomarker which directly affects the treatment choice of physicians and predict the prognosis of the patients treated with chemotherapy. Although assessment of general status is important especially in elderly patients, the assessment could be subjective and systematic scoring of geriatric analysis or fit status were too complicated to perform in clinical practice. sCONUT is a simple, objective and useful nutritional prognostic score that can be easily assessed in clinical practice and have a great potential as the indicator for choosing appropriate induction therapy in elderly AML patients. Our study has some limitations. Our cohort has limited sample size, lack of data about the consolidation therapy, and some patients lack biochemical data due to questionnaire-based retrospective data collection. Our real-world cohort includes heterogeneous patients with various comorbidities. It is obvious that comorbidities would have a huge impact on the nutritional status. We did not assess background complications resulting in poor nutritional status in each patient. Further investigation for comorbidity is needed in the future studies. At least, it was demonstrated that high sCONUT score at diagnosis is a poor prognostic factor in elderly AML patients and this is a notable result which can lead to future investigation.

In conclusion, we report that the prognostic risk classification based on AML disease status using NCCN Guidelines 2017 and new assessment scoring of patients’ nutritional status based on the sCONUT score can easily stratify elderly patients with newly diagnosed AML.

## Supplementary information

Supplementary Information 1.
