# Supplementary material for: A novel nutritional index “simplified CONUT” and the disease risk index independently stratify prognosis of elderly patients with acute myeloid leukemia
Source: Sci Rep. 2020 Nov 10;10:19400. doi: 10.1038/s41598-020-76250-8 (PMC7655799; doi:10.1038/s41598-020-76250-8)
Supplement: Supplementary file 1 — Supplementary Information 1. [file 41598_2020_76250_MOESM1_ESM.pdf]

Supplementary information

A novel nutritional index “simplified CONUT” and the disease risk index independently stratify prognosis of elderly patients with acute myeloid leukemia

Hajime Senjo[1], Masahiro Onozawa[1], Daisuke Hidaka[1], Shota Yokoyama[1], Satoshi Yamamoto[2], Yutaka Tsutsumi[3], Yoshihito Haseyama[4], Takahiro Nagashima[5], Akio Mori[6], Shuichi Ota[7], Hajime Sakai[8], Toshimichi Ishihara[9], Takuto Miyagishima[10], Yasutaka Kakinoki[11], Mitsutoshi Kurosawa[12], Hajime Kobayashi[13], Hiroshi Iwasaki[14], Daigo Hashimoto[1], Takeshi Kondo[6] and Takanori Teshima[1].

[1] Department of Hematology, Faculty of Medicine, Hokkaido University, Sapporo, Japan
[2] Department of Hematology, Sapporo City General Hospital, Sapporo, Japan
[3] Department of Hematology, Hakodate Municipal Hospital, Hakodate, Japan
[4] Department of Hematology, Tonan Hospital, Sapporo, Japan
[5] Department of Hematology, Japanese Red Cross Kitami Hospital, Kitami, Japan
[6] Blood Disorders Center, Aiiku Hospital, Sapporo, Japan
[7] Department of Hematology, Sapporo Hokuyu Hospital, Sapporo, Japan
[8] Department of Hematology, Teine Keijinkai Hospital, Sapporo, Japan
[9] Department of Hematology, Kin-ikyo Chuo Hospital, Sapporo, Japan
[10] Department of Hematology, Kushiro Rosai Hospital, Kushiro, Japan
[11] Department of Hematology, Asahikawa City Hospital, Asahikawa, Japan
[12] Department of Hematology, Hokkaido Cancer Center, Sapporo, Japan
[13] Department of Hematology, Obihiro Kosei General Hospital, Obihiro, Japan
[14] Department of Hematology, Sapporo Kosei General Hospital, Sapporo, Japan

Correspondence Author
Hajime Senjo
Department of Hematology, Faculty of Medicine, Hokkaido University, Sapporo, Japan
N15 W7, Kita-ku, Sapporo, 060-8638, Japan
Telephone: 81-11-865-0111, FAX: 81-11-865-0201
E-mail: hajimesenjo@gmail.com

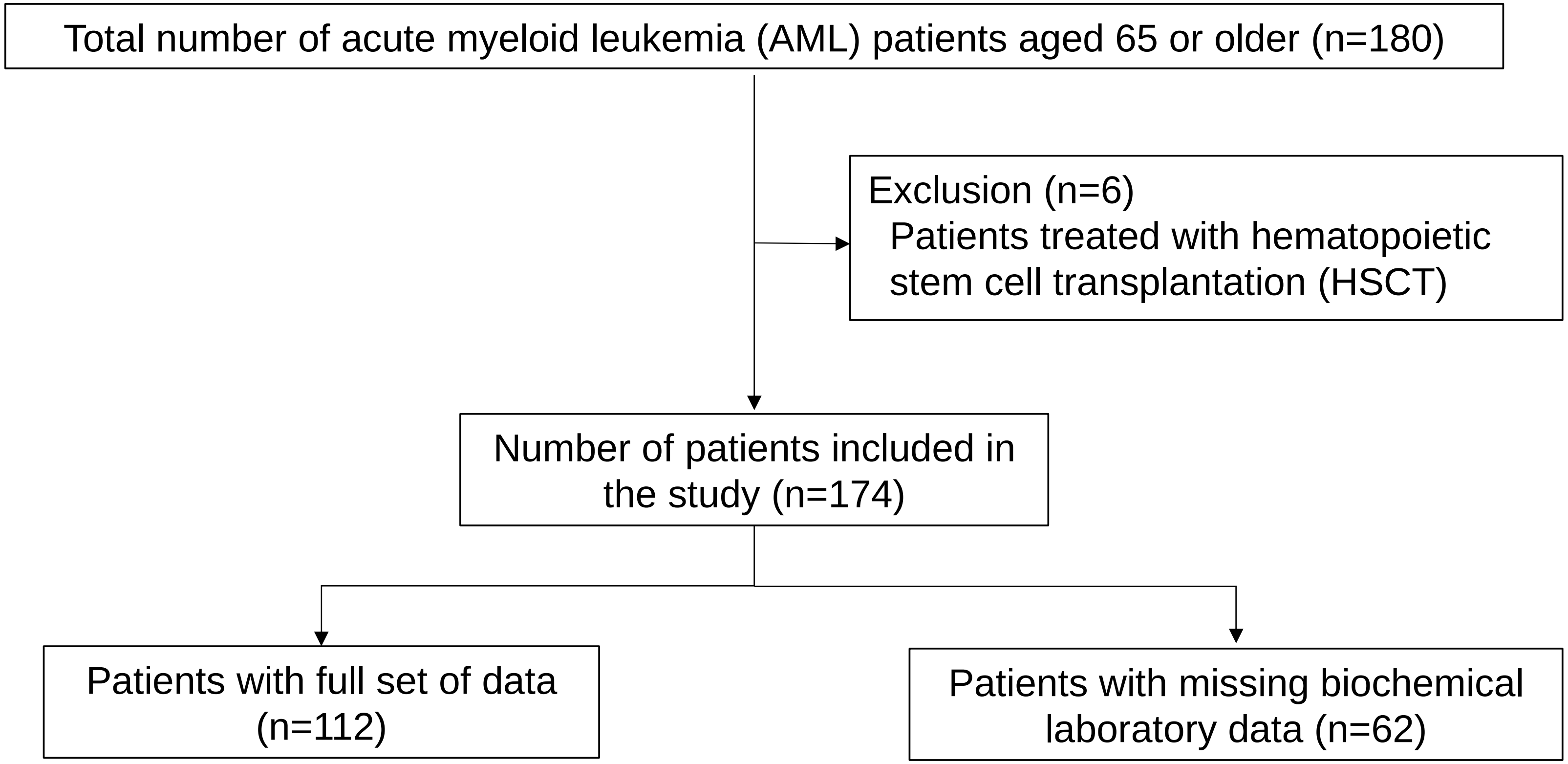

Fig S1. Study consort diagram of the current study

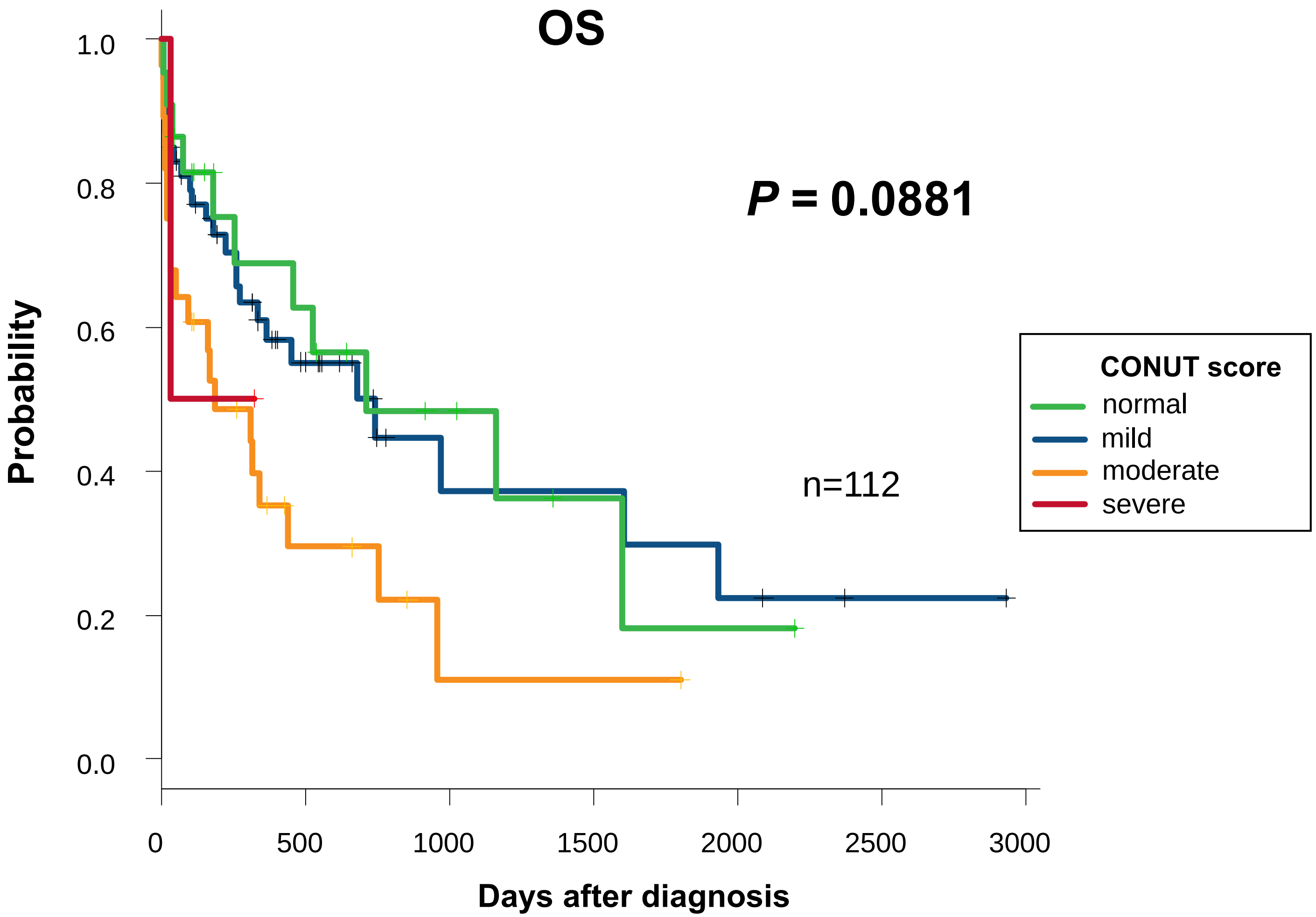

Fig S2. Kaplan-Meier plots of OS according to the classical CONUT score.

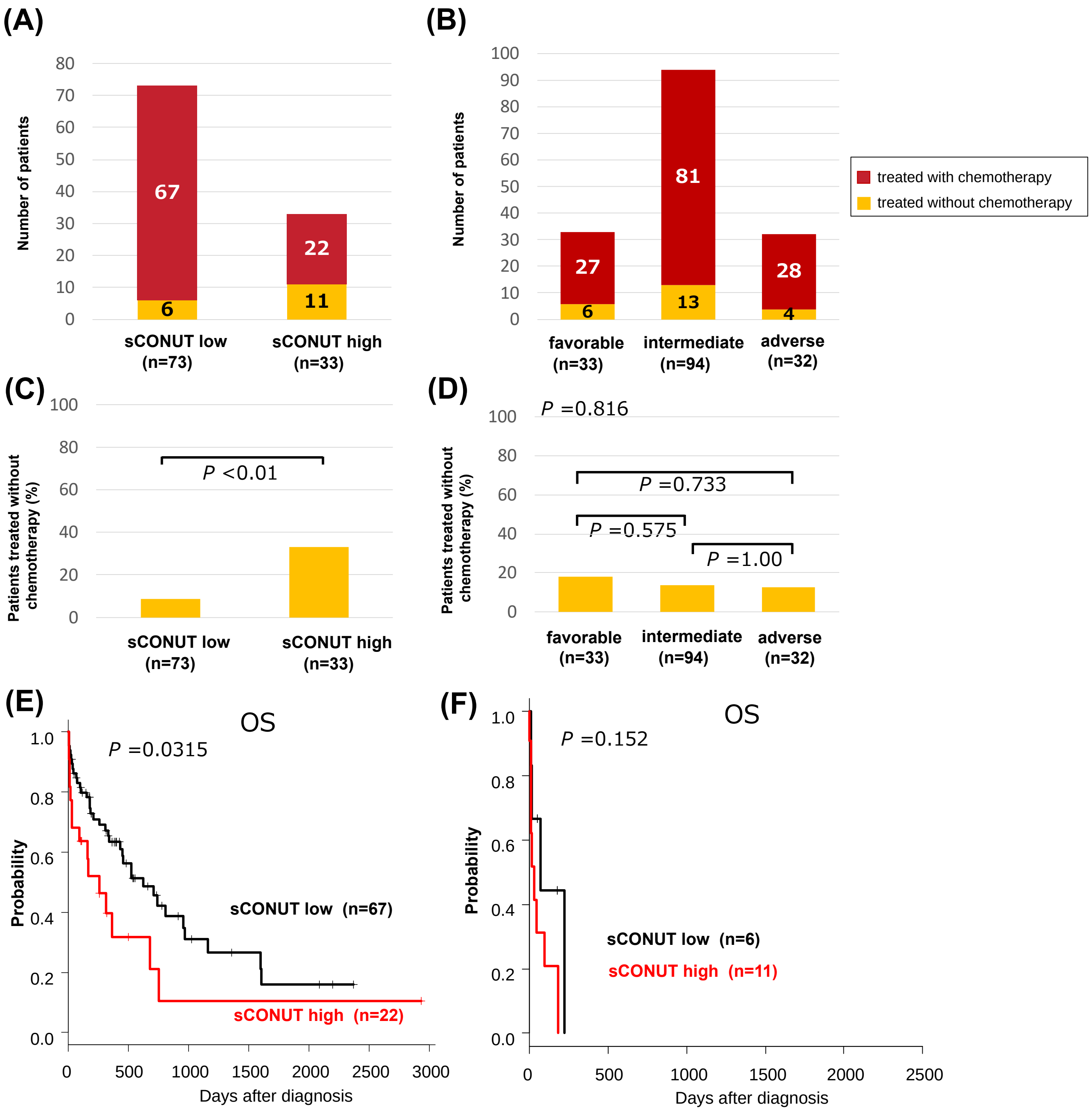

Fig S3. (A,B) The number of patients treated with/without chemotherapy in **(A)** sCONUT high/low groups and **(B)** NCCN2017 favorable/intermediate/adverse groups. **(C,D)** The proportions of patients treated without chemotherapy in **(C)** sCONUT high/low groups and **(D)** NCCN2017 favorable/intermediate/adverse groups. **(E,F)** Kaplan-Meier plots of OS according to sCONUT score of patients treated **(E)** with chemotherapy or **(F)** without chemotherapy.
